# Does pericapsular nerve group block have limited analgesia at the initial post-operative period? Systematic review and meta-analysis

**DOI:** 10.1007/s00540-022-03129-5

**Published:** 2022-11-07

**Authors:** Ahmed Farag, Nada Ibrahim Hendi, Rehab Adel Diab

**Affiliations:** 1 Faculty of Medicine, Misr University for Science and Technology, Giza, Egypt; 2 Faculty of Medicine, Ain Shams University, Cairo, Egypt; 3 Faculty of Medicine, Al-Azhar University, Cairo, Egypt

**Keywords:** Pericapsular nerve group block, PENG block, Hip regional anaesthesia, Systematic review, Meta-analysis

## Abstract

Perioperative pain management related to hip surgical procedures is challenging. Pericapsular nerve group (PENG) block is a novel technique that is assumed to provide better analgesia in addition to its post-operative motor-sparing effect. In this review, we aim to assess the safety and efficacy of PENG block on pain management in patients undergoing hip surgeries. Fifteen clinical trials with a total of 837 patients were included. Pain scores favored PENG group when compared to FICB group (fascia iliaca compartment block) or analgesics-only group, but the difference could only be detected in the early post-operative period. Afterward, it seems to lose its superiority, and no difference could be detected. In addition, cumulative opioid consumption favored PENG group at 24 h but not at 48 h. Regarding patients’ satisfaction, our analysis showed results favoring PENG group, but there was no difference in time to first opioid or length of hospital stay. The incidence of vomiting was lower in PENG, but there was no difference in the incidence of nausea, pruritis, and dizziness. PENG provides better analgesia and lower opioid consumption in the initial post-operative period. Current evidence is not enough, and further high-quality randomized controlled trials with larger sample sizes are required.

**Supplementary Information:**

The online version contains supplementary material available at 10.1007/s00540-022-03129-5.

## Introduction

Hip surgeries are one of the most frequently performed orthopedic procedures nowadays [1]. Perioperative pain, related to these hip surgical procedures, is a major issue that requires attention because it can lead to a wide range of complications, morbidities, and poor overall patient satisfaction [2]. It has a negative impact not only on immediate surgical outcomes but also on long-term prognosis and patients’ quality of life. Persistent pain is found to be associated with worse outcomes and puts the patient at a higher risk of delirium, disturbed cognitive function, sleep disturbance, and anxiety [3]. Moreover, pain hinders physical rehabilitation; thus, leading to poor recovery, prolonged hospital stay, and incredibly increasing the cost. It also leads to delayed mobilization with all the complications that come along with it such as thromboembolic manifestations [4]. Taking into consideration, hip surgical procedures are more frequently done under subarachnoid blocks which makes post-operative pain management more challenging [5, 6]. That is the reason why multimodal analgesia is brought into practice.

Previously, systemic opioids were a mainstay in pain management in the critical perioperative period. However, they cause a lot of adverse effects such as sedation, respiratory depression, nausea, vomiting, constipation, and urinary retention [7]. Moreover, its administration relies on self-reporting of pain and asking for analgesia which is influenced by the probable pre-operative cognitive impairment associated with the condition. That is why different techniques of regional nerve block have emerged to reduce the need for opioids thus sparing its adverse effects. Most of these regional anesthetic techniques target the anterior hip capsule as it has the most sensory innervation of the hip joint. Anterior hip capsule is mainly innervated by the obturator nerve (ON), femoral nerve (FN), and accessory obturator nerves (AON) [8, 9].

Based on a recent anatomical study of the hip capsule innervation, high articular branches of the femoral nerve—which originate cranially to the inguinal ligament—play a major role in the sensory innervation of the anterior hip capsule [8]. Thus, infra-inguinal techniques such as femoral nerve block (FNB) or fascia iliaca compartment block (FICB) have a minimal effect on these branches which leads to insufficient analgesia [10]. Besides, fascia iliaca compartment block (FICB) could not produce evidence of blocking articular branches of the obturator nerve supplying the anterior hip capsule [11]. Additionally, there is an associated muscle weakness due to motor block which increases the risk of post-operative falls and limits early mobilization of the patients [12, 13].

In 2018, the pericapsular nerve group (PENG) block was first described by Girón-Arango et al. [14] as an ultrasound-guided single-injection of local anesthetics targeting the musculofascial plane. This is performed by placing the ultrasound probe in a transverse plane over the anterior inferior iliac spine (AIIS). The probe is then aligned with the pubic ramus by rotating counterclockwise for 45 degrees. Finally, the tip of the needle is inserted to reach the musculofascial plane between the pubic ramus posteriorly and the psoas tendon anteriorly. Local anesthetics are then injected to spread throughout the plane. Unlike other nerve block techniques, PENG block technique targets the articular branches of ON, FN and AON which is assumed to provide better analgesia. Additionally, it was reported that it achieves pain reduction without affecting motor function. Hence, the interest is rapidly growing as this motor-sparing effect helps with early ambulation and better recovery.

In this review, we aim to systematically summarize the existing literature and synthesize evidence on the safety and efficacy of PENG compared to other multimodal analgesic protocols in terms of reduction in pain scores, the need for extra analgesics, and the incidence of complications.

## Methods

### Study registration

The protocol was registered in PROSPERO database (CRD42022339838). This systematic review was performed according to PRISMA statement guidelines, and all steps were done in strict adherence to the Cochrane Handbook of Systematic Reviews and Meta-analysis (Version 5.1.0).

### Criteria for considering studies for this review

We included studies with the following criteria:

Population: patients with hip pathologies undergoing surgical procedures for treatment.

Intervention: pericapsular nerve group (PENG) block.

Comparator: other multimodal analgesic protocols.

Outcome: at least one of the following outcomes must have been reported in the included study (pain score, muscle weakness, opioid consumption, time to the first opioid, length of hospital stay, patient satisfaction, and incidence of complications).

Study design: randomized controlled trial (RCTs).

We excluded studies that did not match these criteria or were not written in the English language. In addition, conference abstracts and protocols were excluded.

### Literature search strategy

We searched the following medical electronic databases: PubMed, Science Direct, WHO Global Health Library, Scopus, and Cochrane Library, all through May 2022. We employed the following keywords: (Hip AND “pericapsular nerve group block” AND “pain management”). The detailed search strategy is attached to the online resource (Table S1). No restrictions or filters were employed.

### Selection of studies

Two subsequent steps were followed to screen the search results for eligibility by two independent reviewers: (1) Title and abstract screening for studies matching the inclusion criteria, and (2) Full-text articles of eligible abstracts were retrieved and screened for eligibility.

### Data extraction

Three authors (AF, NH, RD) extracted the data independently using an online data extraction form. The extracted data includes the following: (1) summary of included studies; (2) baseline characteristics of the study population; (3) risk of bias domains; and (4) study outcomes including pain score, muscle Weakness, post-operative opioid consumption, time to first opioid, length of hospital stay, patient satisfaction score, and complications. Disagreements were resolved by consensus.

### Quality assessment

The quality of the retrieved RCTs was assessed according to the Cochrane handbook of systematic reviews of interventions 5.1.0. We used the quality assessment table provided in (part 2, Chapter 8.5) of the same book. Quality assessment was done by two authors independently. The Cochrane risk of bias assessment tool includes the following domains: sequence generation (selection bias); allocation sequence concealment (selection bias); blinding of participants and personnel (performance bias); blinding of outcome assessment (detection bias); incomplete outcome data (attrition bias); selective outcome reporting (reporting bias); and other potential sources of bias. The authors’ judgment is categorized as ‘Low risk’, ‘High risk’, or ‘Unclear’.

Also, the quality of evidence for all the outcomes was assessed using the GRADE (grading of recommendations, assessment, development, and evaluation). Risk of bias assessment; inconsistency (based on the *I*^2^ statistic); indirectness (resulting from differences in the population of interest, interventions compared, the outcome); imprecision (based on 95% confidence intervals and sample size); and publication bias (asymmetry of the contour enhanced funnel plot and egger's test estimation).

### Measures of treatment effect

The primary outcome was pain score; measured by numeric rating scale (NRS) scores or Visual Analogue Scale (VAS) scores. Other outcomes were opioid consumption, time to the first opioid, length of hospital stay, patient satisfaction, and complications such as (vomiting, nausea, pruritus and dizziness).

### Data synthesis

Effect size and standard error of pain score, post-operative opioid consumption, time to the first opioid, length of hospital stay, and patient satisfaction score were pooled as standardized mean differences (SMDs) in a generic inverse variance model while complications were pooled as relative risk (RR) in a random effect model using the Mantel–Haenszel (M–H) method. We used a random effect model due to the clinical heterogeneity of included studies attributed to different treatment strategies. We used Review Manager 5.4 for Windows.

### Subgroup analysis

Because included studies compared PENG block technique vs different control groups; we performed a subgroup analysis to stratify the control group on pain score. Further analysis was performed based on the time of assessment comparing PENG to fascia iliaca compartment block (FICB) and PENG to analgesics-only.

### Assessment of heterogeneity

Heterogeneity was assessed by visual inspection of the forest plots and measured by *I* square and Chi-Square tests. Significant heterogeneity was defined as (*P* value < 0.1) for chi-square test of heterogeneity. While *I* square test was used to quantify the magnitude of heterogeneity according to recommendations of the Cochrane Handbook of Systematic reviews and meta-analysis.

### Publication bias

For the assessment of publication bias, the pooled effect estimate was plotted against its SE in a funnel plot generated by Jamovi 2.3.13 software. The existence of publication bias was determined by the degree of the figure’s symmetry.

## Results

Our primary literature search in May 2022 retrieved a total of *996 records* from online databases (science direct, PubMed, Scopus, Cochrane library, and WHO Global Health Library). After full-text screening. Fourteen papers were eligible to be included in our study.

**Fig. 1:**
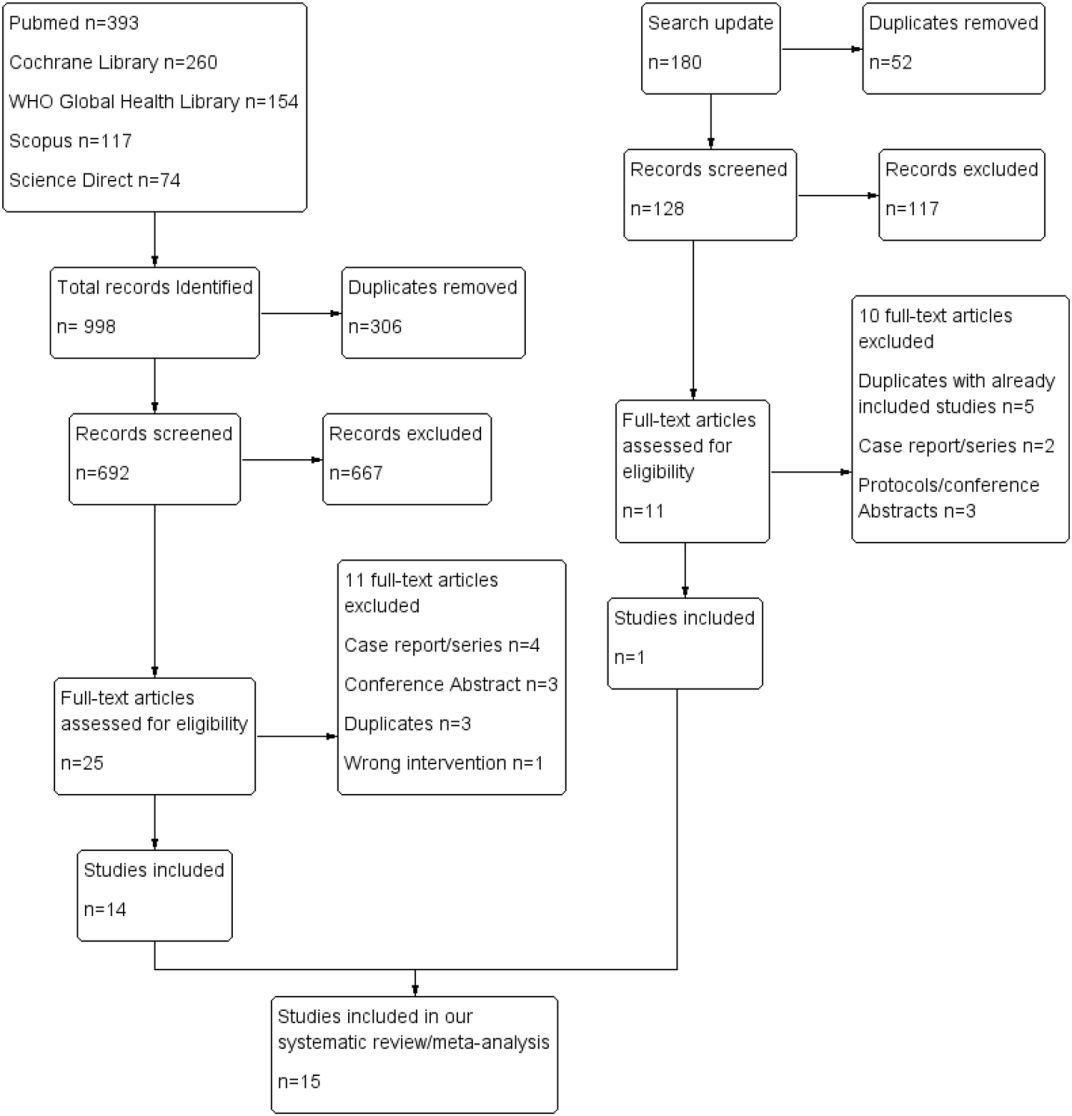
(PRISMA) flow chart representing the search and selection process

We updated our search in August 2022 retrieving a total of 180 records. After screening and applying eligibility criteria, one study [15] was found to be eligible. Reasons for the exclusion of full-text articles are shown in the online resource (Table S2). A detailed description of the literature search and selection process is shown in (PRISMA flow diagram, Fig. 1). A total of 15 studies were included in our final systematic review/meta-analysis. Also, we manually screened references of the included studies. A total of 229 references were screened, and none of them were eligible to be included, which has left us with 15 randomized controlled trials representing 837 patients. Baseline characteristics and summary of the included studies are shown in Table 1 and Table 2, respectively.

**Table 1:** Baseline characteristics

References	Country	Sample size	Intervention	Sex M/F	Age	BMI	Weight	ASA classification (I/II/III/IV)	Type of surgery	Duration of surgery (Mins)
Hua [24]	China	24	PENG	14/10	74 (± 7)	24 (± 3)	N/A	0/6/18/0	HA/THA	133 (± 13)
		24	FICB	13/11	74 (± 8)	23 (± 4)		0/7/17/0		129 (± 19)
Pascarella [22]	Italy	30	PENG	16/14	66.4 (± 12.4)	29.2 (± 5.2)	N/A	3/17/10/0	THA	104 (± 17)
		30	Conventional analgesic therapy	17/13	66.7 (± 8.6)	28 (± 3.9)		4/15/11/0		107 (± 22)
Lin [25]	Australia	30	PENG	14/16	77.2 (± 11.6)	24.16 (± 6.22)	65.6 (± 17.8)	1/5/22/2	Gamma nail/Cannulated screw/HA/THR	N/A
		30	FNB	7/23	79.7 (± 11.5)	23.8 (± 4.82)	65.0 (± 15.7)	2/3/21/4		
Choi [23]	Korea	27	PENG	14/13	60.5 (± 18.39)	25.8 (± 3.0)	N/A	5/15/7/0	THA	68 (± 1.43)
		27	S-FICB	16/11	62 (± 14.87)	25.0 (± 3.9)		2/22/3/0		70.3 (± 15.65)
Zheng [19]	Korea	25	PENG	15/10	60.0 (± 10.9)	N/A	64 (± 10.21)	0/23/2/0	THA	97 (± 14.15)
		27	Periarticular infiltration	16/11	63.0 (± 11.7)		62.8 (± 12.83)	0/20/7/0		99.16 (± 18.39)
Abd-Elhalim [18]	Egypt	30	PENG	18/12	51.2 (± 4.9)	N/A	73.0 (± 6.1)	0/21/9/0	N/A	64.9 (± 12.0)
		30	Intravenous Fentanyl	17/13	50.5 (± 5.3)		74.4 (± 6.1)	0/17/13/0		64.4 (± 11.3)
Alrefaey [17]	Egypt	30	PENG	N/A	54 (± 11)	27 (± 5)	72 (± 9)	N/A	N/A	N/A
		30	Conventional analgesic therapy		57 (± 8)	25 (± 8)	74 (± 13)			
Aliste [16]	Chile	20	PENG	7/13	56.8 (± 13)	27.6 (± 3.8)	N/A	9/11/0/0	THA	74.9 (± 28)
		20	S-FICB	7/13	59.6 (± 9.2)	28.4 (± 4.6)		6/13/1/0		73.5 (± 17.3)
Jadon [27]	India	33	PENG	13/20	70.39 (± 11.45)	30.15 (± 3.76)	71.78 (± 6.24)	23/5/5/0	N/A	N/A
		33	S-FICB	14/19	67.87 (± 13.12)	29.5 (± 3.67)	70.98 (± 8.23)	24/5/4/0		
Shankar [26]	India	30	PENG	20/10	53.58 (± 19.95)	N/A	60.8 (± 13.7)	19/6/5/0	N/A	N/A
		30	FICB	21/19	49.54 (± 21.61)		62.7 (± 10.4)	20/7/3/0		
Senthil [28]	India	20	PENG	10/10	53.9 (± 9.9)	N/A	N/A	9/11/0/0	DHS/Proximal femur nailing	N/A
		20	FICB	12/8	52.5 (± 9.8)			7/13/0/0		
Zheng [29]	China	34	PENG	12/22	63 (57–69)	23.3 (21.7–24.9)	56 (51–61)	27/3/4/0	THA	113 (97–129)
		36	Placebo	15/21	64 (59–68)	23.6 (22.5–24.6)	58 (55–62)	28/1/7/0		106 (94–118)
Mosaffaa [20]	Iran	30	PENG	22/8	53 (± 16.46)	N/A	71.6 (± 6.97)	N/A	DHS, Gamma nail, and screws	N/A
		22	FICB	16/6	50 (± 13.63)		74.81 (± 3.56)			
Scanaliato [21]	USA	32	PENG	15/17	37.9 (± 4.4)	27.03 (± 6.7)	N/A	N/A	Hip Arthroscopy	N/A
		32	LPB	12/20	38.6 (± 3.6)	29.93 (± 10.1)				
Güllüpınar [15]	Turkey	18	PENG	16/9	78.6 (± 10.51)	24.67 (± 4.97)	N/A	N/A	N/A	N/A
		21	Conventional analgesic therapy	14/7	72.5 (± 20.11)	25.16 (± 3.05)				

Data are presented as mean (± SD), median (IQR)

*PENG* Pericapsular nerve group block, *FICB* Fascia iliaca compartment block, *FNB* Femoral nerve block, *LPB* Lumber plexus block, *S-FICB* Supra-Inguinal Fascia Iliaca Compartment Block, *HA* Hemiarthroplasty, *THA* Total Hip Arthroplasty, *DHS* Dynamic hip screw, *ST* Subtrochanteric fracture, *IT* Intertrochanteric fracture, *THR* Total hip replacement

**Table 2:** Summary of the included studies

References	Design	Groups	S.S	Time of intervention and type of anesthesia	Injectate	Indication	Outcomes		
							Pain score	Opioid	Motor sparing
Hua [24]	Single center, Single-blind, RCT	PENG	24	Before SA	20 ml of 0.4% ropivacaine	Facilitate positioning/POP	P.G	No diff	P.G
		FICB	24		30 ml of 0.4% ropivacaine				
Pascarella [22]	Single center, Single-blind, RCT	PENG	30	Prior to operation after SA	20 ml of 0.375% ropivacaine	POP	P.G	P.G	P.G
		Conventional analgesic therapy	30		–				
Lin [25]	Single center, Double-blinded, RCT	PENG	30	15–45 min prior to operation (GA/SA)	20 ml of 0.75% ropivacaine	POP	P.G	No diff	P.G
		FNB	30		20 ml of 0.75% ropivacaine				
Choi [23]	Single center, Double-blinded, RCT	PENG	29	Prior to operation after GA	20 ml of 0.2% ropivacaine + epinephrine 1:200,000	POP	P.G	No diff	No diff
		S-FICB	29		30 ml of 0.2% ropivacaine + epinephrine 1:200,000				
Zheng [19]	Single center, Single-blind, RCT	PENG	30	Prior to operation after SA	30 ml of 0.5% ropivacaine	POP	No diff	No diff	N/A
		Periarticular infiltration	30		20 ml of ropivacaine 0.75%, ketorolac 2 ml, and epinephrine 1 ml mixed with normal saline				
Abd-Elhalim [18]	Multicenter, Single-blinded, RCT	PENG	30	Before SA	20 ml of 0.125% bupivacaine	Facilitate positioning/POP	P.G	P.G	N/A
		Intravenous Fentanyl	30		Intravenous 0.5 μg/kg body weight fentanyl				
Alrefaey [17]	Single center, Single-blinded, RCT	PENG	30	Before SA	20 ml of 0.25% bupivacaine	Facilitate positioning	N/A	N/A	N/A
		Conventional analgesic therapy	30		-				
Aliste [16]	Single center, RCT	PENG	20	After operation (SA)	20 ml of 0.5% adrenalized levobupivacaine	POP	No diff	No diff	P.G
		S-FICB	20		40 ml of 0.25% adrenalized levobupivacaine				
Jadon [27]	Single center, Double-blinded, RCT	PENG	33	Before SA	N/A	Facilitate positioning/POP	P.G	No diff	N/A
		S-FICB	33		N/A				
Shankar [26]	Double-blinded, RCT	PENG	30	Before SA	25 ml of 0.25% ropivacaine	Facilitate positioning/POP	P.G	No diff	N/A
		FICB	30		25 ml of 0.25% ropivacaine				
Senthil [28]	Double-blinded, RCT	PENG	20	After operation (SA)	30 ml 0.25% levobupivacaine with 4 mg dexamethasone	POP	P.G	P.G	P.G
		FICB	20		30 ml of 0.25% Levobupivacaine and 4 mg dexamethasone				
Zheng [29]	Single center, Double-blinded, RCT	PENG	34	Prior to operation Before GA	20 ml of 0.5% ropivacaine	POP	P.G	No diff	No diff
		Placebo	36		20 ml 0.9% saline				
Mosaffaa [20]	Single center, Double-blinded, RCT	PENG	30	Before SA	3 ml/kg ropivacaine 0.5% (a maximum of 40 ml)	POP	P.G	P.G	N/A
		FICB	22		3 ml/kg of ropivacaine 0.5% (a maximum of 40 ml)				
Scanaliato [21]	Multicenter, Single-Blinded, RCT	PENG	32	Prior to operation after GA	30 ml of ropivacaine + 12 mg of morphine	POP	No diff	No diff	N/A
		LPB	32		40 ml of 0.375% ropivacaine + 4 mg of dexamethasone				
Güllüpınar [15]	Single-center, RCT	PENG	18	In the ED	20 ml of bupivacaine 0.25%	Pain control	P.G	N/A	N/A
		Conventional analgesic therapy	21		–				

*S.S* Sample size, *SA* Spinal anesthesia, *GA* general anesthesia, *PENG* Pericapsular nerve group block, *FICB* Fascia iliaca compartment block, *FNB* Femoral nerve block, *LPB* Lumber plexus block, *S-FICB* Supra-Inguinal Fascia Iliaca Compartment Block, *POP* post-operative pain, *P.G* favor PENG group, *No diff* No difference, *ED* Emergency department

**Fig. 2:**
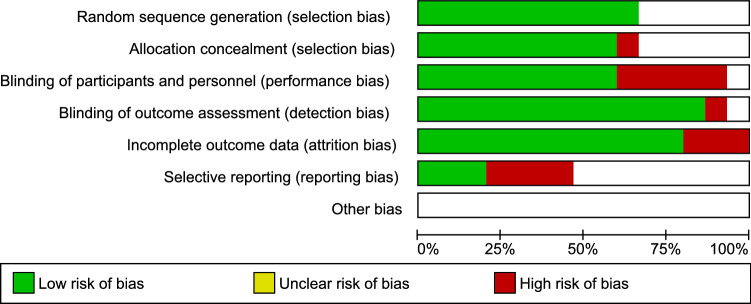
Risk of bias graph for included studies

**Fig. 3:**
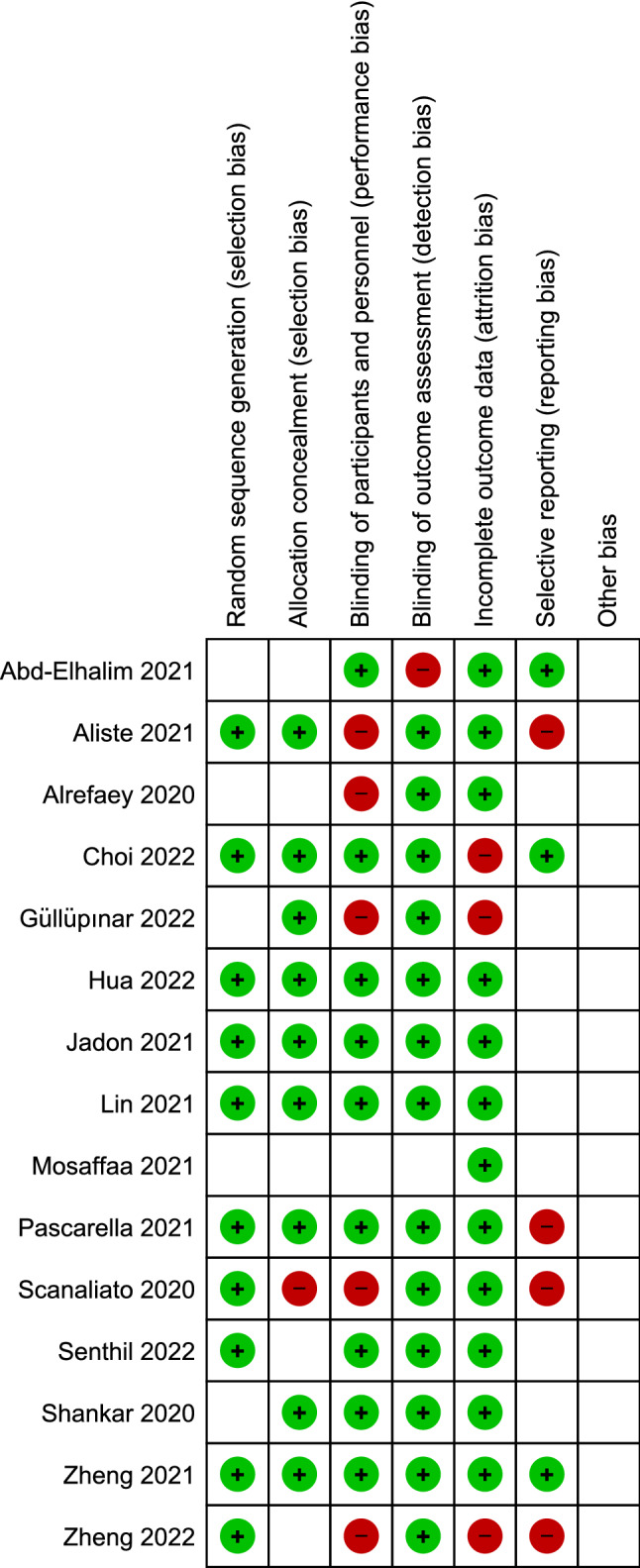
Risk of bias summary according to the Cochrane risk of bias assessment tool; risk of bias domains include mainly (selection bias, performance bias, detection bias, attrition bias, and reporting bias)

**Fig. 4:**
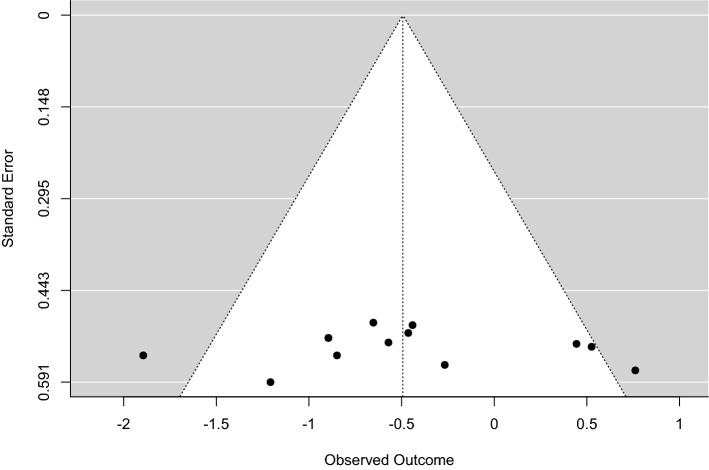
Funnel plot of the primary outcome

Based on the Cochrane risk of the bias assessment tool, the quality of the included studies ranged from low to high quality with seven studies of low quality [15–21]; two of moderate quality [22, 23]; and six high-quality studies [24–29]. Risk of bias graph and summary of quality assessment domains of included studies are shown in Figs. 2 and 3 respectively. Authors’ judgments with justifications are shown in the online resource (Table S3). Regarding reporting bias, there was no reporting bias detected as the funnel plot of our primary outcome did not express asymmetry Fig. 4.

### Pain score [primary outcome]

**Fig. 5:**
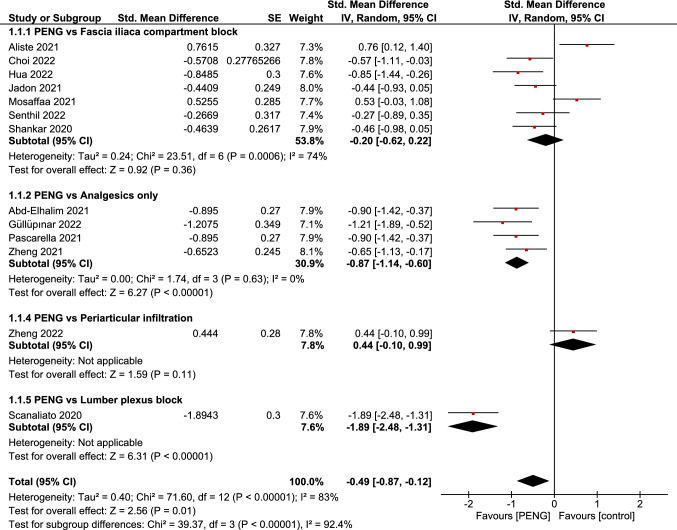
Forest plots of standardized mean difference of dynamic pain scores measured around 30 min postoperatively comparing between PENG block and other controls. The red diamonds represent the effect of individual studies, and the vertical lines show the corresponding 95% confidence intervals (CI). The black diamond reflects the overall or summary effect. The outer edges of the diamonds represent the CIs

The overall pooled analysis of dynamic pain scores measured around 30 min postoperatively by VAS or NRS showed a statistically significant difference favoring PENG group (SMD =  − 0.49; 95% CI = [− 0.87, − 0.12]; *p* = 0.01; very low grade of evidence; Fig. 5). There was significant total heterogeneity among the pooled studies (*p* =  < 0.00001; *I*^2^ = 83%).

Stratification analysis of the control group on postoperative pain scores showed a statistically significant difference in favor of PENG when compared to lumbar plexus block or analgesics-only (SMD = -0.87; 95% CI = [-1.14, -0.60]; P < 0.00001; very low grade of evidence; Fig. 5) but showed no statistically significant difference when compared to periarticular infiltration or FICB (SMD = -0.20; 95% CI = [-0.62, 0.22]; *P* = 0.36; very low grade of evidence; Fig. 5). Heterogeneity in different subgroups was significant only in FICB subgroup (*P* = 0.0006; *I*^2^ = 74%).

In another scenario, we excluded studies with a high risk of bias [15, 16, 18–21]. The overall SMD did not change significantly (SMD -0.59; 95% CI [-0.79 to -0.39]; *p* > 0.00001; low grade of evidence; Fig. not shown). Moreover, heterogeneity was resolved (*p* = 0.72; *I*^2^ = 0%). Consistency of the overall effect estimates, despite the removal of the high risk of bias studies, confirms that the results obtained from our analysis are statistically robust.

By omitting studies with a high risk of bias, subgroups effect estimates favored PENG group when compared to both subgroups remaining which are FICB subgroup (SMD =  − 0.52; 95% CI = [0.76, − 0.27]; *P* < 0.0001; low grade of evidence); and analgesics-only subgroup (SMD =  − 0.76; 95% CI = [− 1.12, − 0.41]; *P* < 0.0001; low grade of evidence). In this scenario, no heterogeneity was detected in any of the subgroups.

### Pain score (PENG vs FICB) at different time points

**Fig. 6:**
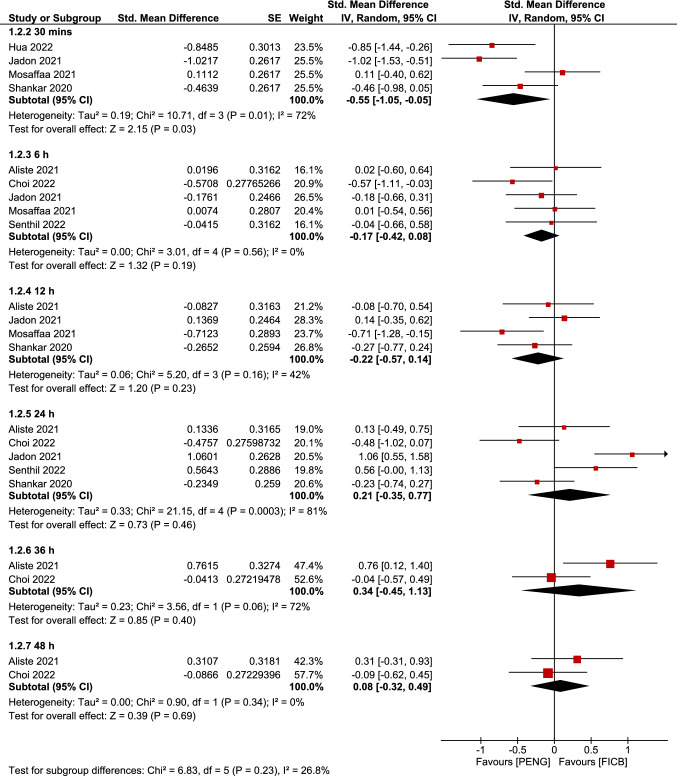
Forest plots of standardized mean difference of dynamic pain scores measured at different time points postoperatively comparing between PENG block and FICB. The red diamonds represent the effect of individual studies, and the vertical lines show the corresponding 95% confidence intervals (CI). The black diamond reflects the overall or summary effect. The outer edges of the diamonds represent the CIs

Analysis comparing PENG and FICB groups was performed to stratify the time of assessment on post-operative pain scores. The overall pooled analysis of subgroups between PENG group and FICB group showed a statistically significant difference favoring PENG group at 30 min postoperatively (SMD =  − 0.55; 95% CI = [− 1.05, − 0.05]; *p* = 0.03; moderate grade of evidence; Fig. 6) but not at 6 h, 12 h, 24 h 36 h, and 48 h which did not favor either of the two groups. There was significant heterogeneity among the pooled studies which was resolved or lowered to a moderate level by omitting studies with a high risk of bias [16, 20] (Fig. not shown). Despite the removal of the high risk of bias studies, the results did not change which confirms that the results obtained are statistically robust.

### Pain score (PENG vs analgesics-only) at different time points

**Fig. 7:**
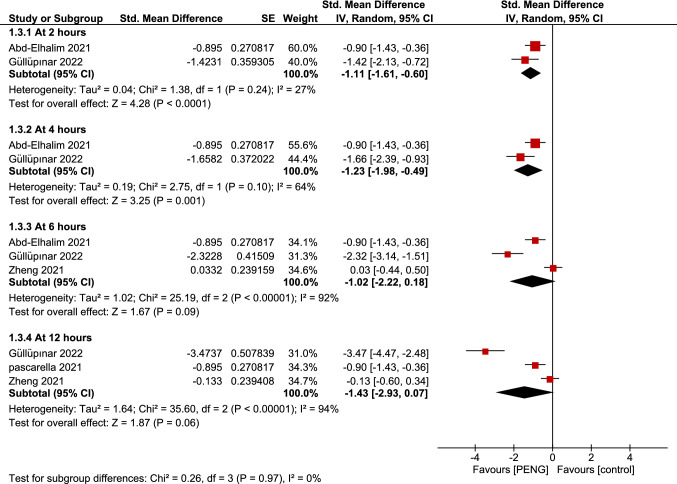
Forest plots of standardized mean difference of dynamic pain scores measured at different time points postoperatively comparing between PENG block and analgesics-only. The red diamonds represent the effect of individual studies, and the vertical lines show the corresponding 95% confidence intervals (CI). The black diamond reflects the overall or summary effect. The outer edges of the diamonds represent the CIs

Analysis comparing PENG and analgesics-only groups was performed to stratify the time of assessment on post-operative pain scores. The overall pooled analysis of subgroups between PENG group and FICB group showed a statistically significant difference favoring PENG group at 2 h and 4 h postoperatively (SMD =  − 1.11; 95% CI = [− 1.61, − 0.60]; *p* > 0.0001; very low grade of evidence; Fig. 7) (SMD =  − 1.23; 95% CI = [− 1.98, − 0.49]; *p* = 0.001; very low grade of evidence; Fig. 7), respectively, with low to moderate heterogeneity. However, no difference could be detected at 6 h and 12 h.

### Opioid consumption

**Fig. 8:**
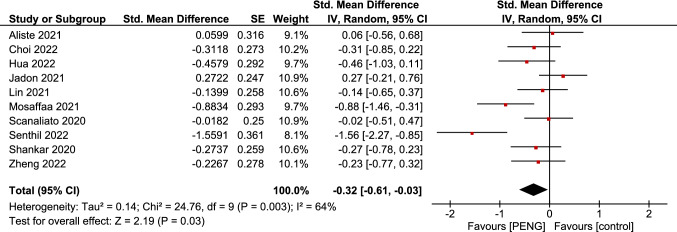
Forest plots of standardized mean difference of post-operative cumulative opioid consumption at 24 h. The red diamonds represent the effect of individual studies, and the vertical lines show the corresponding 95% confidence intervals (CI). The black diamond reflects the overall or summary effect. The outer edges of the diamonds represent the CIs

Ten of the included studies, representing 536 patients, reported opioid consumption at 24 h postoperatively. The overall pooled SMD favored PENG group in terms of lower opioid consumption in the first 24 h (SMD =  − 0.32; 95% CI = [− 0.61, − 0.03]; *p* = 0.03; very low grade of evidence; Fig. 8). There was heterogeneity among the pooled studies (*p* = 0.003; *I*^2^ = 64%). Heterogeneity is attributed to the fact that most of the studies used different doses and types of analgesics at different pain thresholds.

On the other hand, eight of the included studies, representing 448 patients, reported opioid consumption at 48 h postoperatively. The overall pooled SMD did not favor either of the two groups (SMD =  − 0.25; 95% CI = [− 0.54, 0.04]; *p* = 0.09; very low grade of evidence; supplementary file. Fig. S1). There was heterogeneity among the pooled studies (*p* = 0.02; *I*^2^ = 57%).

### Time to first opioid

Three of the included studies, representing 178 patients, reported time to the first opioid postoperatively. The overall pooled SMD did not favor either of the two groups (SMD = 0.34; 95% CI = [− 0.79, 1.48]; *p* = 0.55; very low grade of evidence; supplementary file. Fig. S2). There was heterogeneity among the pooled studies (*p* = 0.00001; *I*^2^ = 93%).

### Length of hospital stay

Three of the included studies, representing 154 patients, reported time to the first opioid postoperatively. The overall pooled SMD did not favor either of the two groups (SMD =  − 0.10; 95% CI = [− 0.41, 0.22]; *p* = 0.55; moderate grade of evidence; supplementary file. Fig. S3). The pooled studies were homogeneous (*p* = 0.69; *I*^2^ = 0%).

### Patient satisfaction

Seven of the included studies, representing 420 patients, reported overall patient satisfaction. The overall pooled SMD favored PENG group in terms of higher overall patient satisfaction (SMD = 0.63; 95% CI = [0.27, 1.00]; *p* = 0.0007; low grade of evidence; supplementary file. Fig. S4). There was heterogeneity among the pooled studies (*p* = 0.08; *I*^2^ = 47%).

### Complications

#### Vomiting

Two studies, representing 130 patients, reported an incidence of vomiting postoperatively. The overall pooled RR favored PENG group in terms of lower incidence of vomiting (RR = 0.32; 95% CI = [0.13, 0.80]; *p* = 0.01; low grade of evidence; supplementary file. Fig. S5-a). The pooled studies were homogeneous (*p* = 0.63; *I*^2^ = 0%).

#### Nausea

Seven studies, representing 400 patients, reported an incidence of nausea postoperatively. The overall pooled RR of incidence of nausea did not favor either of the two groups (RR = 0.91; 95% CI = [0.51, 1.62]; *p* = 0.75; Very low grade of evidence; supplementary file. Fig. S5-b). There was heterogeneity among the pooled studies (*p* = 0.21; *I*^2^ = 28%).

#### Pruritis

Three studies, representing 152 patients, reported incidence of purities postoperatively. The overall pooled RR of incidence of purities did not favor either of the two groups (RR = 0.95; 95% CI = [0.10, 9.28]; *p* = 0.96; Very low grade of evidence; supplementary file. Fig. S5-c). There was heterogeneity among the pooled studies (*p* = 0.19; *I*^2^ = 40%).

#### Dizziness

Two studies, representing 112 patients, reported an incidence of dizziness postoperatively. The overall pooled RR of incidence of dizziness did not favor either of the two groups (RR = 1.04; 95% CI = [0.11, 9.71]; *p* = 0.97; low grade of evidence; supplementary file. Fig. S5-d). The pooled studies were homogeneous (*p* = 0.32; *I*^2^ = 0%).

## Discussion

PENG block was originally developed to control pain and provide analgesia in hip-fracture patients [14]. One of the main advantages of this technique is the supine position, which is suitable for patients suffering from acute or chronic pain due to hip fractures. In addition, it has a motor-sparing effect which is achieved by targeting only the sensory articular branches of the femoral nerve (FN) and accessory obturator nerve [14]. Applications of the technique are expanding; it was recently used not only as an alternative regional anesthesia technique for acute pain in hip fracture patients but also for analgesia after elective hip surgeries [30, 31]. Recent case reports used the technique for other anesthetic purposes beyond the hip joint capsule such as vein ligation and stripping [32]; however, it was highly criticized and described as “undesired effect” by Girón-Arango et al. [33].

Our meta-analysis showed that PENG block was associated with superior analgesic effects as compared to other analgesic protocols such as FICB and analgesics-only. Dynamic pain scores were significantly better favoring PENG group in the immediate post-operative period. Our results go along with the results of all papers included except for three papers with a high risk of bias [16, 21, 29]. One of them [29] is the only paper that compared PENG to periarticular infiltration (PAI) and reported no difference detected regarding pain scores which may indicate non-inferiority of the PENG technique. PAI not only targets anterior hip capsule innervation, but also the posterior hip capsule which gives an advantage regarding pain control. Concerning the clinical aspects of both interventions; PAI depends on direct infiltration of injectable materials around the joint which can be done easily intraoperatively, but it is not possible after surgery in adverse to PENG group which is a plane block technique that is possible to be applied preoperatively and postoperatively [34].

The difference in pain scores, when PENG compared to FICB or analgesics-only, was only significant during the early post-operative period (less than 6 h). However, no significant difference in pain reduction was detected at longer periods of follow up which indicates that the effect of the block diminishes over time. A table of the exact pain scores extracted is presented in the online resource (Table S4) for further details. A recent case report by Singh et al. [35] reported a successful case of prolonged analgesic duration achieved by PENG block for 3 days through the use of a catheter. However, the clinical efficacy of continuous or multidose PENG block still needs further studies to investigate.

All clinical trials retrieved from our literature search used PENG block technique for analgesia of hip fracture patients except one [21] used it for patients undergoing hip arthroscopy to perform femoroplasty, labral repair, and reconstruction. All studies included used PENG block technique for scheduled elective surgeries except five: one study [15] used it for emergency cases and four studies [16, 18, 20, 24] did not report any data on that point. Studies included were highly different in terms of the time of intervention, main indication, and volume of injectate. Regarding the time of intervention and main indication; five studies [17, 18, 24, 26, 27] used PENG technique to position patients for spinal anesthesia in addition to post-operative pain control; seven studies [19–23, 25, 29] used it preoperatively for post-operative pain control; two [16, 28] used it postoperatively for pain management; and one study [15] used it for analgesia in the emergency department with no association to the operation time. Regarding the dose of injectate; all studies reported using 20 ml of volume injectate to perform PENG block except six studies; three of them [19, 21, 28] used 30 ml; and one [26] used 25 ml of volume. Further details of substances used are provided in Table 2.

Although Girón-Arango et al. [33] stated that using more than 20 ml for PENG block may cause undesirable motor blocks which was agreed by some case reports [36, 37]; we could not notice any association between increasing volume of injectate and muscle weakness which can be explained by two main reasons; first, studies that used high volumes did not report any data on muscle strength; second, small sample sizes of the studies included may fade out the association.

Extracted data regarding quadriceps muscle weakness were not suitable for meta-analysis because different quantitative and qualitative scales were used to report; although, most of the included papers reported less muscle weakness in PENG group. Only two studies [23, 29] reported no difference regarding muscle weakness which was probably concealed in the early post-operative period due to general anesthesia used in both. The optimum concentration of local anesthesia to achieve sufficient pain relief without causing quadriceps weakness is an aspect that would have to be investigated further; but, to the best of our knowledge, 10–20 mL of injectate would cover the articular branches of the obturator nerve as suggested by a previous dye injection cadaveric study [38]. Greater volumes than 20 ml or any iatrogenic errors, such as intramuscular injection, may result in a motor block.

Our meta-analysis showed the statistical superiority of PENG in lowering the need for opioid consumption during the first 24 h post-procedure but not during the first 48 h which also suggests that the analgesic effect of PENG wears off over time. Three studies [20, 22, 27] reported time to the first opioid; two of them [20, 22] reported results favoring PENG over FICB and conventional analgesic therapy respectively, but our meta-analysis showed no statistical significance. The same applies to the length of hospital stay; three studies [16, 22, 23] reported data regarding hospital stay duration; two of them [22, 23] reported results favoring PENG over conventional analgesic therapy and SI-FICB respectively, but our meta-analysis showed no statistical significance.

Patient Satisfaction score was reported in seven studies [18, 19, 21, 23, 25, 26, 29] and all of them favored PENG technique. Although it’s a subjective outcome; it may reflect the strong analgesic effect of the technique. None of the included studies representing 837 patients reported adverse reactions related to intervention application such as puncture site infection and hematoma which is highly suggestive that the application of the PENG block technique may be safer than other regional hip anesthetic techniques. Considering the fact that PENG block technique targets an area close to the hip joint; aseptic measures should be strictly followed throughout the procedure to avoid hip joint infection.

Other complications were reported and analyzed as follows; vomiting was reported in two studies [18, 29], and our analysis showed statistically significant results favoring PENG in terms of less incidence of vomiting. In addition, nausea was reported in seven studies [16, 18, 19, 21–23, 29]; pruritis was reported in three studies [16, 18, 19]; dizziness was reported in two studies [19, 22], and none of them showed any significant difference.

### Limitations

A high degree of imprecision regarding some outcomes may exist due to the small sample sizes of included trials. The included studies compared PENG block technique against limited number of comparators. Instead, different multimodal analgesia protocols should have been compared to recognize the pros and cons of this newly developed technique.

Many included studies reported very few data on pain scores at different time points. Therefore, our primary outcome was a pain score at a time point “around 30 min”, which cannot alone reflect the efficacy of the technique. In addition, most of the studies did not meticulously report on opioid consumption in terms of doses, types, and duration of consumption. In addition, no clinical trials could be retrieved from the literature that were conducted to test continuous PENG block using catheter techniques or continuous blocks which is hypothesized to provide a long-lasting analgesic effect [39]. Same applies to combining PENG block with sciatic block or local infiltration analgesia techniques which is hypothesized to provide a complete hip capsule [40, 41].

Our findings should be interpreted in light of the quality of evidence, ranging from moderate to very low. The included studies varied in several areas such as block performance, volume of injectate, time of outcome measurement, analgesic protocols in the control group, and type of rescue opioids. Hereby, A high risk of random error may exist.

## Conclusion

In conclusion, our meta-analysis shows that PENG block technique can provide better pain control which leads to less opioid consumption. As a result, higher overall satisfaction is obtained from the patients receiving PENG block. However, PENG block loses its superiority over time which suggests the need for modification regarding the number of injections. Length of hospital stay and time to the first opioid are not different. Regarding complications, PENG block is associated with a lower incidence of post-operative vomiting, but the incidence of nausea, pruritis, and dizziness are not different. Current evidence is insufficient to confirm the safety and efficacy of PENG block technique. Therefore, further well-designed trials with larger sample sizes are needed.

## Supplementary Information

Below is the link to the electronic supplementary material.

Supplementary file1 (DOCX 109 KB)

## Data Availability

The datasets are available from the corresponding author upon reasonable request.
