# Supplementary material for: Does pericapsular nerve group block have limited analgesia at the initial post-operative period? Systematic review and meta-analysis
Source: J Anesth. 2022 Nov 7;37(1):138–53. doi: 10.1007/s00540-022-03129-5 (PMC9870839; doi:10.1007/s00540-022-03129-5)
Supplement: Supplementary file 1 — Supplementary file1 (DOCX 109 KB) [file 540_2022_3129_MOESM1_ESM.docx]

*Table S1: Search strategy*

| Database | Search Terms |
| --- | --- |
| PubMed | (Hip OR Trochanteric OR Intertrochanteric OR Subtrochanteric OR Subcapital OR femoral neck) AND (Fracture OR surgery OR replacement OR arthroplasty OR fractures OR perioperative OR postoperative OR preoperative OR "after surgery") AND (PENG OR "pericapsular nerve group" OR "pericapsular nerve" OR pericapsular OR "autonomic nerve" OR "peripheral nerve") AND (block OR anaesthesia OR anesthesia OR anaesthetic OR anesthetic OR "pain management" OR numbness OR analgesia OR analgesic) |
| WHO Global Health Library | (Hip OR trochanteric OR subtrochanteric OR Intertrochanteric OR subcapital OR "femoral neck") AND ("pericapsular nerve" OR PENG OR "pericapsular nerve group" OR pericapsular) |
| Scopus | (Hip OR Trochanteric OR Intertrochanteric OR Subtrochanteric OR (femoral neck)) AND (Fracture OR surgery OR replacement OR arthroplasty OR fractures OR perioperative OR postoperative OR preoperative OR (after surgery)) AND (PENG OR (pericapsular nerve group) OR (pericapsular nerve) OR pericapsular) AND (block OR anesthesia OR anesthesia OR anesthetic OR anesthetic OR (pain management) OR numbness OR analgesia OR analgesic) |
| Science Direct | Hip AND "pericapsular nerve" |
| Cochrane Library | (Hip OR Trochanteric OR Intertrochanteric OR Subtrochanteric OR Subcapital OR femoral neck) AND (Fracture OR surgery OR replacement OR arthroplasty OR fractures OR perioperative OR postoperative OR preoperative OR "after surgery") AND (PENG OR "pericapsular nerve group" OR "pericapsular nerve" OR pericapsular OR "autonomic nerve" OR "peripheral nerve") AND (block OR anaesthesia OR anesthesia OR anaesthetic OR anesthetic OR "pain management" OR numbness OR analgesia OR analgesic) |

*Table S2: Reasons for Excluded Studies*

| **Study title** | **Author, year** | **Reason for exclusion** |
| --- | --- | --- |
| A comparison of hip flexor motor function and analgesia associated with pericapsular nerve group blockade and femoral nerve blockade for patients undergoing neck of femur fracture surgery | Muircheartaigh, 2022 | Conference Abstract |
| Comparing the efficacy of pericapsular nerve group block (peng) block versus supra-inguinal fascia iliaca block (fib) in hip arthroplasty | Vamshi, 2021 | Conference Abstract |
| Impact of the pericapsular nerve group (PENG) block on postoperative analgesia and functional recovery following total hip arthroplasty: a randomised, observer-masked, controlled trial | Pascarella, 2021 | Duplicate |
| Pericapsular nerve group (PENG) block provides improved short-term analgesia compared with the femoral nerve block in hip fracture surgery: a single-center double-blinded randomized comparative trial | Lin, 2021 | Duplicate |
| Ultrasound guided peri-articular infiltration analgesia for patients undergoing hip arthrosopy: a prospective, randomized, double-blind, placebo-controlled study | Sinha, 2015 | Not PENG block technique |
| PENG block and LIA as a possible anesthesia technique for total hip arthroplasty. | Sandri, 2020 | Case series |
| Pericapsular Nerve Group (PENG) block for perioperative pain control in hip arthroscopy. | Orozco, 2020 | Case series |
| Pediatric use of Pericapsular Nerve Group (PENG) block for hip surgical procedures. | Orozco, 2019 | Case report |
| Lumbar plexus block versus peri-capsular injection for hip arthroscopy: a single-blinded randomized controlled trial | Polmear, 2021 | Conference Abstract |
| Randomized comparison between pericapsular nerve group (PENG) block and suprainguinal fascia iliaca block for total hip arthroplasty | Aliste, 2021 | Duplicate |
| Pericapsular nerve group (PENG) block: A feasibility study of landmark based technique | Jadon, 2020 | Case series |
| **search update** | | |
| Combined pericapsular nerve group and lateral femoral cutaneous nerve blocks for hip fracture in a polytraumatized patient | Valoriani, 2022 | Case report |
| Preoperative pericapsular nerve group (PENG) block for total hip arthroplasty: a randomized, placebo-controlled trial | Zheng, 2022 | Duplicate |
| Continuous pericapsular nerve group (PENG) block through an elastomeric infusion system, associated with the lateral cutaneous nerve block of the thigh for total hip arthroplasty | Costa, 2022 | Case report |
| Ultrasound Guided Pericapsular Nerve Group Block Versus Quadratus Lumborum Block | Wahdan, 2022 | Study protocol |
| Pericapsular Nerve Group (PENG) Block versus Supra-Inguinal Fascia Iliaca Compartment Block for Total Hip Arthroplasty: A Randomized Clinical Trial | Choi, 2022 | Duplicate |
| Evaluation of Pericapsular Nerve Group (PENG) Block for Analgesic Effect in Elderly Patients with Femoral Neck Fracture Undergoing Hip Arthroplasty | Hua, 2022 | Duplicate |
| PENG Block Plus Lateral Femoral Cutaneous Block for Posterolateral-approached Total Hip Arthroplasty | Pascarella, 2022 | Study protocol |
| Pericapsular Nerve Group (PENG) Block Combined With Periarticular Multimodal Drug Injection (PMDI) Versus Isolated PMDI for Pain Management After Total Hip Arthroplasty: a Randomized Controlled Trial | Park, 2022 | Study protocol |
| Comparison of Pericapsular Nerve Group Block versus Fascia  Iliaca Compartment Block as Postoperative Pain Management  in Hip Fracture Surgeries | Senthil, 2022 | Duplicate |
| Effectiveness of pericapsular nerve group block with ultrasonography in patients diagnosed with hip fracture in the emergency department | Güllüpınar, 2022 | Duplicate |

*Table S3: Support for ROB judgement*

|  | Random sequence generation | Allocation concealment | Blinding of participants and personnel | Blinding of outcome assessment | Incomplete outcome data | Selective reporting | Other bias |
| --- | --- | --- | --- | --- | --- | --- | --- |
| Hua 2022 | A computerized random number generator was used | Sealed opaque envelopes were used | Patients were blinded to group allocation. | Another investigator, independent of group assignment, was responsible for collecting postoperative data. | No loss of follow up | No protocol was found | - |
| Pascarella 2021 | A computerized random number generator was used | Consecutively numbered, sealed, opaque envelopes were used | Patients were blinded to group allocation. | Outcome assessors were blinded | No loss of follow up | Change in pain scale [NRS instead of VAS] | - |
| Lin 2021 | An online randomization computer generator was used (www.sealed-envelope.com) | Sealed envelopes were used | Patients were blinded to group allocation. | Members of the surgical, anesthetic, Acute Pain Service (APS), study and nursing staff were blinded for the intervention. | Intention to treat analysis was performed | No protocol was found | - |
| Choi 2022 | A computerized random number generator was used | Sealed opaque envelopes were used | Patients were blinded to the group assignment during the study period. | All outcomes and perioperative data were collected by an investigator blinded to the group allocation. | 13% lost to follow-up (<5%), balanced reasons but could be related to the intervention | As the protocol | - |
| Zheng 2022 | A computerized random number generator was used | The random table was uploaded to the REDCap software and was accessible only to the researcher who prepared the study drug but it is not mentioned that this researcher is not involved in the study. | Patients were not blinded as the authors believed it was unethical to use a placebo injection. therefore, the outcome, pain score, will be affected | The outcome accessor was blinded to group allocation. | 6% lost and unbalanced reasons | Different endpoint of the 1ry outcome (24 h instead of 48 h for pain score) | - |
| Abd-Elhalim 2021 | Closed envelope method was used | Not mentioned that the envelope is sealed or opaque | Patients were blinded to the group assignment | It is not mentioned whether the clinician who performed the block was involved in the follow-up of outcomes or not | the number allocated is the same as that analyzed | As the protocol | - |
| Mosaffaa 2021 | Not mentioned | Not mentioned | The method of blinding is not mentioned | Not mentioned | No loss to follow up | No protocol was found | - |
| Alrefaey 2020 | A closed envelope technique was used | Not mentioned that the envelope is sealed or opaque | The patient cannot be blinded as one group did not receive any block. | The attending anesthetist was blinded for the patient group | No loss to follow up | No protocol was found | - |
| Aliste 2021 | A computerized random number generator was used | A sealed, opaque envelope technique was used. | Patients were not blinded as they could guess their group allocation. | The primary outcomes assessors were blinded | No loss to follow up | Did not report an outcome that was planned to be reported (urinary retention). | - |
| Jadon 2021 | A computerized random number generator was used | Sequentially numbered opaque envelopes were used. | Patients were unaware of the group and the procedure performed. | All observations were done by another anesthesiologist who was unaware of the regional block performed. | No loss to follow up | No protocol was found | - |
| Shankar 2020 | Not mentioned | Sealed opaque envelope method was used. | The patient and assessor of the visual analog scale were blinded to group allocation. | Assessors of the visual analog scale were blinded to group allocation. | The number randomized is the same analyzed | No protocol was found | - |
| Senthil 2022 | A computerized random number generator was used | Not mentioned | The patient were blinded to the study. | Outcome assessors were blinded | Less than 5% were excluded from the analysis for balanced reasons | No protocol was found | - |
| Zheng 2021 | An online randomization computer generator was used | Sealed, numbered and opaque envelopes were used. | Patients were blinded to group allocation. | The data collection was performed by blinded study investigators. | Only one patient was removed from the study before receiving the intervention | Same as protocol | - |
| Scanaliato 2020 | A simple randomization technique was performed | the list resulting from this process was accessible by the operative surgeon (A.B.W.) and the anesthesiologist (P.S.G.). | Patients were blinded to group allocation. | the PACU staff members responsible for recording the patient’s postoperative pain and medications administered were blinded to the intervention. | No loss to follow up | Change in pain scale | - |
| Güllüpınar 2022 | Not mentioned | A sealed envelope system was used for randomization. | The patient was aware of the procedure and this could affect his reporting of pain score | The physician performing the PENG procedure was blinded to the NRS scores of the patients both pre- and post-procedure. | Unbalanced losses in both groups | No protocol was found | - |

*Table S4: Results of individual studies reporting on pain*

| Study ID | Intervention | Control | Pain score assessment | | | | | |  |
| --- | --- | --- | --- | --- | --- | --- | --- | --- | --- |
|  |  |  | Scale | Time of assessment | Intervention | Control | P-value | |  |
| Hua 2022 | PENG | FICB | VAS | **30 mins** | **2.9 (±0.3)** | **3.2 (±0.4)** | | **-** |  |
|  |  |  |  |  |  |  |  |  |  |
| Abd El-halim 2021 | PENG | IVF | VAS | **2 h** | **1(0–1)** | **0(0–1)** | | **<0.001** |  |
|  |  |  |  | 4 h | 1(1–2) | 1(0–1) | | <0.001 |  |
|  |  |  |  | 6 h | 2(1–2) | 1(1–1) | | <0.001 |  |
|  |  |  |  | 8 h | 2(2–3) | 1(1–2) | | <0.001 |  |
|  |  |  |  | 12 h | 3(2–4) | 2(1–3) | | <0.001 |  |
| Mosaffaa 2021 | PENG | FICB | VAS | 30 min post-anesthesia | 2.93 (±0.78) | 2.82 (±1.22) | | 0.7 |  |
|  |  |  |  |  |  |  |  |  |  |
|  |  |  |  | **At recovery** | **3.47 (±1.04)** | **2.91 (±1.1)** | | **0.073** |  |
|  |  |  |  | 6 h | 3.46 (±1.27) | 3.45 (±1.47) | | 0.97 |  |
|  |  |  |  | 12 h | 3.01 (±1.08) | 3.91 (±1.48) | | 0.021 |  |
| Shankar 2020 | PENG | FICB | VAS | 30 min post block | 0.6 (±0.4) | 2.6 (±1.2) | | <0.001 |  |
|  |  |  |  | **30 min post-operative** | **1.45 (±0.6)** | **1.7 (±0.47)** | | **0.52** |  |
|  |  |  |  | 1 h | 1.55 (±0.69) | 1.8 (±0.41) | | 0.572 |  |
|  |  |  |  | 4 h | 2.46 (±1.07) | 2.16 (±0.91) | | 0.23 |  |
|  |  |  |  | 12 h | 6.14 (±0.95) | 6.4 (±1.01) | | 0.18 |  |
| Senthil 2022 | PENG | FICB | VAS | **2 h** | **0.60 (± 0.940)** | **0.85 (±0.933)** | | **0.404** |  |
|  |  |  |  | 6 h | 1.40 (±0.681) | 1.65 (±8.489) | | 0.791 |  |
|  |  |  |  | 14 h | 1.45 (±0.605) | 1.80 (±0.834) | | 0.137 |  |
|  |  |  |  | 24 h | 1.65 (±0.813) | 1.25 (±0.639) | | 0.092 |  |
| Zheng 2021 | PENG | Placebo | VAS | **At PACU** | **3.3 (±2.7)** | **5.2 (±3.1)** | | **<0.01** |  |
|  |  |  |  | 0-6 h | 4.6 (3.7-5.4) | 4.5 (3.5-5.4) | | 0.89 |  |
|  |  |  |  | 6-24 h | 3.7 (3.0-4.4) | 4.0 (3.2-4.8) | | 0.58 |  |
|  |  |  |  | 24-48 h | 3.1 (2.3-3.8) | 3.3 (2.5-4.1) | | 0.68 |  |
| Pascarella 2021 | PENG | Conventional analgesic therapy | NRS | **0-12 h** | **2.5 (2.0–3.7 [0.0–7.0])** | **5.5 (5.0–7.0 [2.0–8.0])** | | **<0.001** |  |
|  |  |  |  | 12-24 h | 3 (2–4 [0–7]) | 6 (5–6 [2–8]) | | **-** |  |
|  |  |  |  | 24-48 h | 2 (2–4 [0–5]) | 3.0 (2.0–4.7 [0.0–6.0]) | | - |  |
| Choi 2022 | PENG | S-FICB | NRS | **6 h** | **5.8 (±1.55)** | **7 (±2.59)** | | **0.159** |  |
|  |  |  |  | 24 h | 5 (±1.55) | 6 (±2.59) | | 0.116 |  |
|  |  |  |  | 36 h | 2.1 (±1.558) | 2.7 (±2.078) | | >0.999 |  |
|  |  |  |  | 48 h | 4.3 (±2.07) | 4.5 (±2.59) | | 0.298 |  |
| Zheng 2022 | PENG | PAI | NRS | **1 h** | **2.0 (1.0-4.0)** | **1.0 (0.0-2.5)** | | **0.116** |  |
|  |  |  |  | 6 h | 2.0 (1.0-4.0) | 1.0 (0.0-3.0) | | 0.216 |  |
|  |  |  |  | 12 h | 3.0 (1.0-4.0) | 2.0 (1.0-4.0) | | 0.465 |  |
|  |  |  |  | 24 h | 2.0 (1.0-5.0) | 2.0 (1.0-3.5) | | 0.683 |  |
| Aliste 2021 | PENG | S-FICB | NRS | **3 h** | **3 (0–8)** | **1.5 (0–9)** | | **0.021** |  |
|  |  |  |  | 6 h | 3 (0–9) | 3 (0–7) | | 0.951 |  |
|  |  |  |  | 12 h | 2.5 (0–8) | 3 (0–6) | | 0.795 |  |
|  |  |  |  | 24 h | 2 (0–8) | 2 (0–5) | | 0.675 |  |
|  |  |  |  | 36 h | 1.5 (0–9) | 0 (0–5) | | 0.021 |  |
|  |  |  |  | 48 h | 1 (0–5) | 1 (0–5) | | 0.332 |  |
| Jadon 2021 | PENG | S-FICB | NRS | 30 min post block | 4 (1) | 5 (1) | | 0.004 |  |
|  |  |  |  | **4 h** | **1 (1)** | **1 (2)** | | **0.078** |  |
|  |  |  |  | 6 h | 1 (2) | 1 (2) | | 0.477 |  |
|  |  |  |  | 12 h | 4 (0) | 3 (1.5) | | 0.58 |  |
|  |  |  |  | 24 h | 3 (1.5) | 2 (1) | | 0.000* |  |
| Scanaliato 2020 | PENG | LPB | NRS | **30 min post operative** | **2.31 (±0.46)** | **3.25 (±0.53)** | | **0.21** |  |
|  |  |  |  | 60 min | 1.76 (±0.22) | 2.77 (±0.19) | | 0.1 |  |
|  |  |  |  | 90 min | 0.81 (±0.15) | 0.9 (±0.17) | | 0.14 |  |
| Güllüpınar 2022 | PENG | Conventional analgesic therapy | NRS | **30 min** | **3.06 (±1.80)** | **5.24 (±1.81)** | | **0.001** |  |
|  |  |  |  |  |  |  |  |  |  |
|  |  |  |  | 6 h | 0.22 (±0.43) | 4.29 (±2.35) | | 0.001 |  |
|  |  |  |  |  |  |  |  |  |  |
|  |  |  |  | 12 h | 1.28 (±1.41) | 6.33 (±1.49) | | 0.001 |  |
|  |  |  |  |  |  |  |  |  |  |
|  |  |  |  | 24 h | 2.44 (±1.50) | 7.14 (±1.24) | | 0.001 |  |
|  |  |  |  |  |  |  |  |  |  |

Data are presented as mean ± SD, median (IQR) , median(IQR[range])


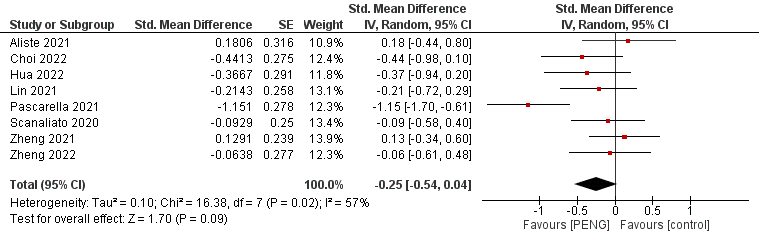


**Fig. S1** Forest plots of standardized mean difference of postoperative cumulative opioid consumption at 48 h


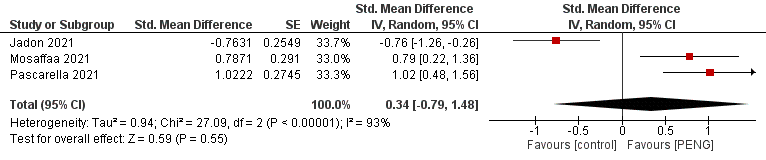
 **Fig. S2** Forest plots of standardized mean difference of duration to first opioid consumption


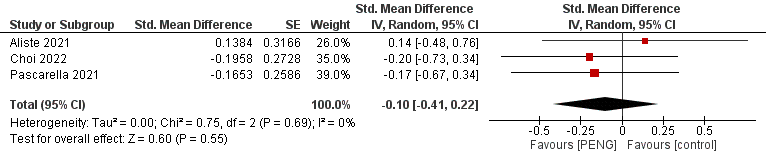


**Fig. S3** Forest plots of standardized mean difference of hospital stay length


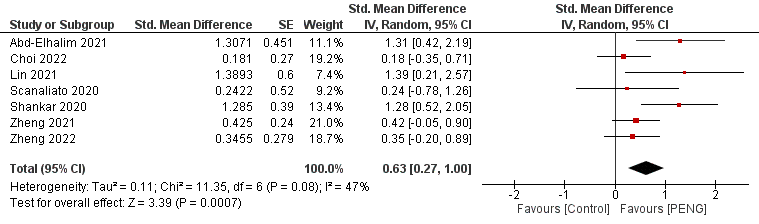


**Fig. S4** Forest plots of standardized mean difference of patients’ satisfaction score


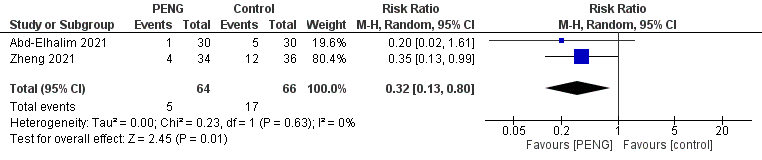


**Fig. S5-a** Forest plot of risk ratio of postoperative vomiting incidence


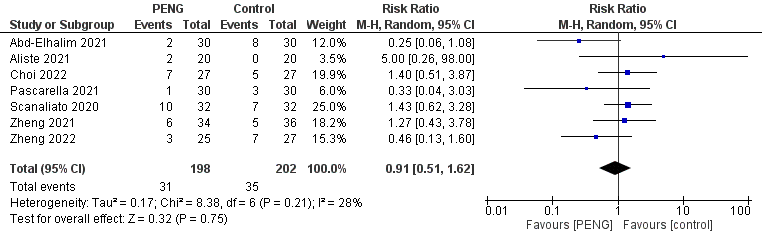


**Fig. S5-b** Forest plot of risk ratio of postoperative nausea incidence


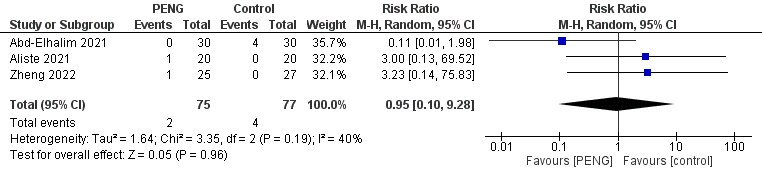
 **Fig. S5-c** Forest plot of risk ratio of postoperative pruritis incidence


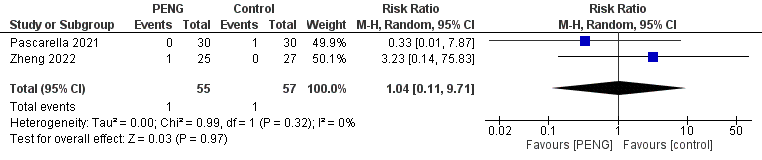


**Fig. S5-d** Forest plot of risk ratio of postoperative dizziness incidence
